# Genetically encoded fluorescent biosensors for GPCR research

**DOI:** 10.3389/fcell.2022.1007893

**Published:** 2022-09-29

**Authors:** Hyunbin Kim, In-Yeop Baek, Jihye Seong

**Affiliations:** ^1^ Brain Science Institute, Korea Institute of Science and Technology (KIST), Seoul, South Korea; ^2^ Division of Bio-Medical Science and Technology, KIST School, Korea University of Science and Technology, Seoul, South Korea; ^3^ Department of Converging Science and Technology, Kyung Hee University, Seoul, South Korea

**Keywords:** GPCR, fluorescent protein, genetically encoded fluorescent biosensor, FRET, circular permutation, BRET, nanobody

## Abstract

G protein-coupled receptors (GPCRs) regulate a wide range of physiological and pathophysiological cellular processes, thus it is important to understand how GPCRs are activated and function in various cellular contexts. In particular, the activation process of GPCRs is dynamically regulated upon various extracellular stimuli, and emerging evidence suggests the subcellular functions of GPCRs at endosomes and other organelles. Therefore, precise monitoring of the GPCR activation process with high spatiotemporal resolution is required to investigate the underlying molecular mechanisms of GPCR functions. In this review, we will introduce genetically encoded fluorescent biosensors that can precisely monitor the real-time GPCR activation process in live cells. The process includes the binding of extracellular GPCR ligands, conformational change of GPCR, recruitment of G proteins or β-arrestin, GPCR internalization and trafficking, and the GPCR-related downstream signaling events. We will introduce fluorescent GPCR biosensors based on a variety of strategies such as fluorescent resonance energy transfer (FRET), bioluminescence resonance energy transfer (BRET), circular permuted fluorescent protein (cpFP), and nanobody. We will discuss the pros and cons of these GPCR biosensors as well as their applications in GPCR research.

## Introduction

G protein-coupled receptor (GPCR) is the biggest receptor family involved in the regulation of diverse cellular processes (Pierce et al., 2002; Lefkowitz, 2013). About 800 members of the GPCR family recognize a variety of ligands, including hormones, lipids, and neurotransmitters, then transfer this diverse information to the cells. GPCRs also sense other external stimuli, such as light, odors, and mechanical signals, and convert them into biochemical signals in the cells. As expected from their diverse functions (Lagerstrom and Schioth, 2008; Wu et al., 2019), GPCRs are major therapeutic targets of more than 30% of FDA-approved drugs (Insel et al., 2019). Thus, it is important to understand how GPCRs are activated and function in various cellular contexts.

**FIGURE 1:**
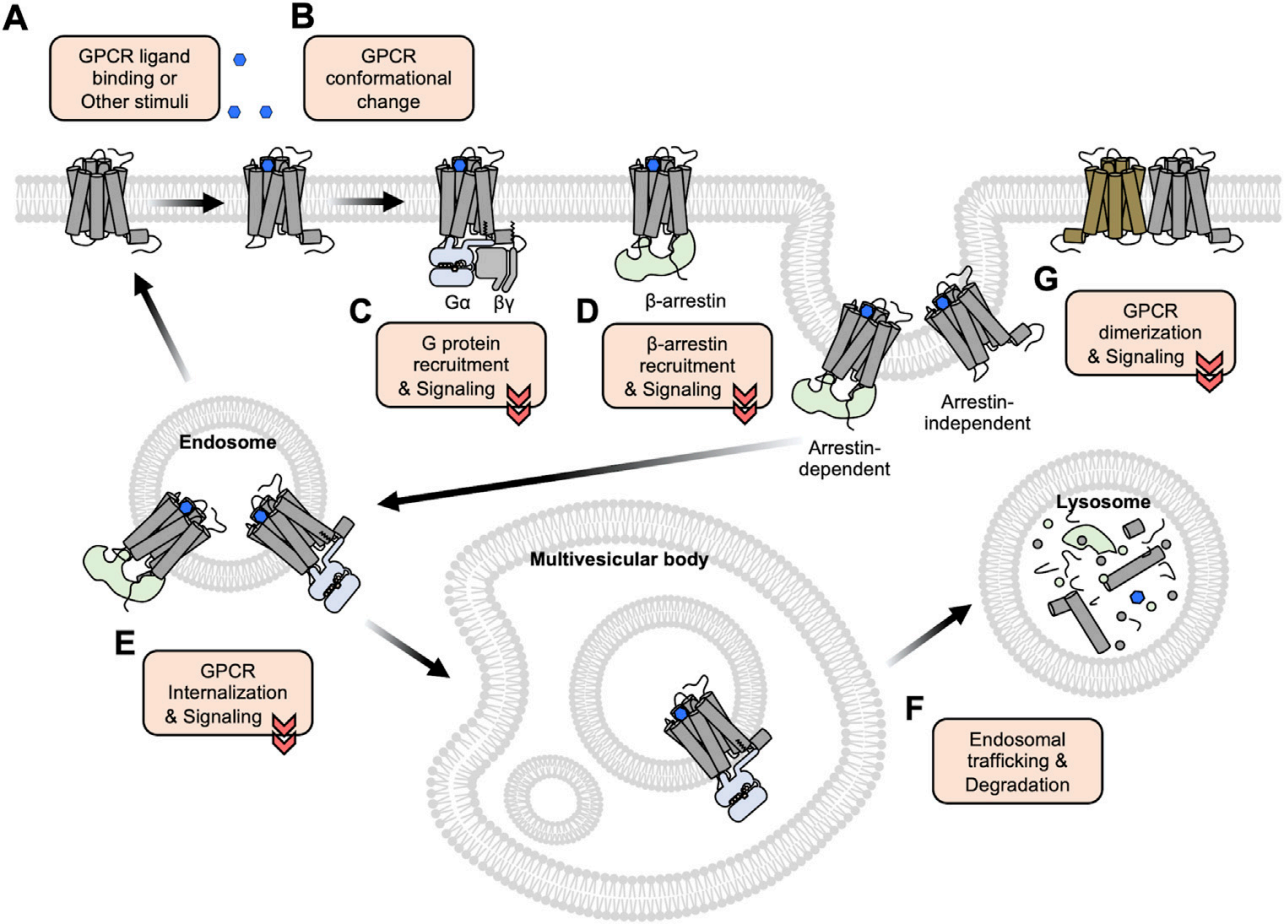
An overview of GPCR activation, trafficking, and degradation. **(A)** GPCRs bind to the extracellular ligands or other stimuli at the plasma membrane. **(B)** The ligand-bound GPCRs change their conformations, in particular between TM5 and TM6. **(C–D)** The conformational changes of the GPCRs induce the recruitment of G proteins **(C)** or β-arrestin **(D)** to initiate the downstream signaling pathways. **(E)** The recruited β-arrestin promotes the internalization of GPCRs via the clathrin-mediated endocytosis pathway. Some GPCRs can maintain their activity and function at the internalized endosomes or subcellular organelles. **(F)** Following the endosomal trafficking pathways, the GPCRs can finally be degraded in the lysosome. **(G)** Some GPCRs form dimers or oligomers. Functional crosstalk between these GPCRs may influence the ligand binding affinity, downstream signaling pathways, and trafficking patterns.

GPCRs are composed of seven transmembrane (TM) domains connected by three extracellular loops (ECLs) and three intracellular loops (ICLs) with an extracellular N-terminus and an intracellular C-terminus (Vassilatis et al., 2003). When a ligand binds to the corresponding GPCR from the extracellular side (Figure 1A), the receptor changes its conformation (Figure 1B), facilitating the interactions with G proteins or β-arrestin at the intracellular side of the plasma membrane (Figure 1C,D) which further mediates the downstream signaling pathways. It has been traditionally believed that GPCRs function at the plasma membrane and lose their activity after internalization, but recent studies show that some GPCRs can keep their activity at the internalized endosomes or subcellular organelles (Eichel and von Zastrow, 2018; Mohammad Nezhady et al., 2020) (Figure 1E). In addition, class C GPCRs function as obligatory dimers and emerging evidence indicates that some of class A GPCRs can be also dimerized or oligomerized, which can affect the ligand binding affinity, downstream signaling pathways, and trafficking patterns (Pin et al., 2007; Lohse, 2010) (Figure 1G). Therefore, it is crucial to investigate the molecular mechanisms of GPCR activation and function at the subcellular levels for the correct understanding of the complex physiology of GPCRs and the pathophysiology of related diseases.

**FIGURE 2:**
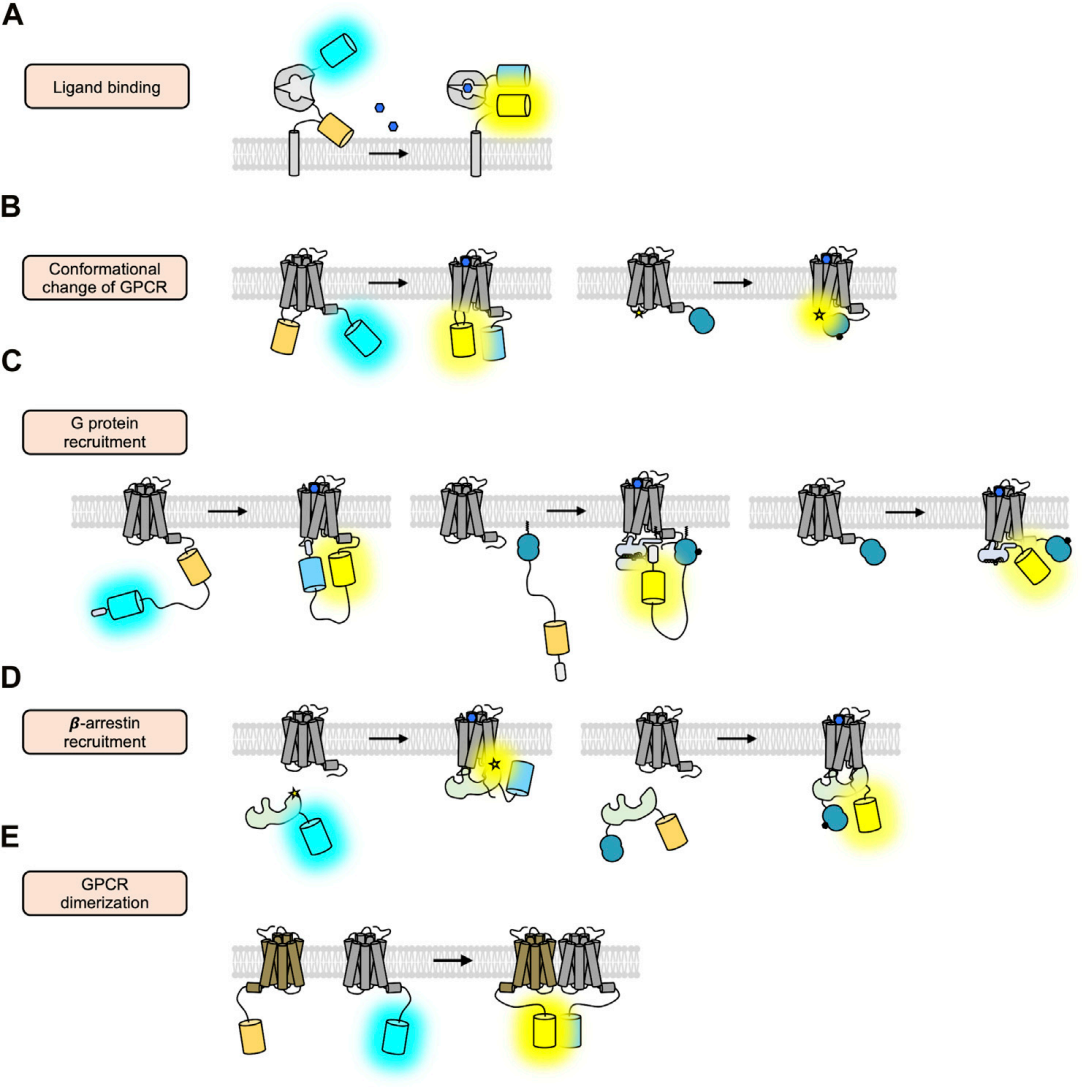
FRET/BRET-based GPCR biosensors. **(A)** Schematic design of the ligand-sensing biosensors based on FRET (Tsien, 2006). The FRET signal between cyan and yellow FP inserted in the N- and C-terminus of the ligand-sensing domain is increased upon ligand binding. **(B)** Schematic design of the FRET/BRET-based biosensors detecting the conformational change of GPCRs (Griffin et al., 1998; Vilardaga et al., 2005). In the right panel, a blue circle represents a luciferase and a star displays FlAsH. **(C)** Schematic design of the FRET/BRET-based biosensors detecting the recruitment of G proteins (Malik et al., 2013; Wan et al., 2018; Maziarz et al., 2020). **(D)** Schematic design of the FRET/BRET-based biosensors detecting the recruitment of β-arrestin (Charest et al., 2005; Nuber et al., 2016). **(E)** Schematic design of the FRET-based biosensors detecting the dimerization of GPCRs (Lukasiewicz et al., 2010).

**FIGURE 3:**
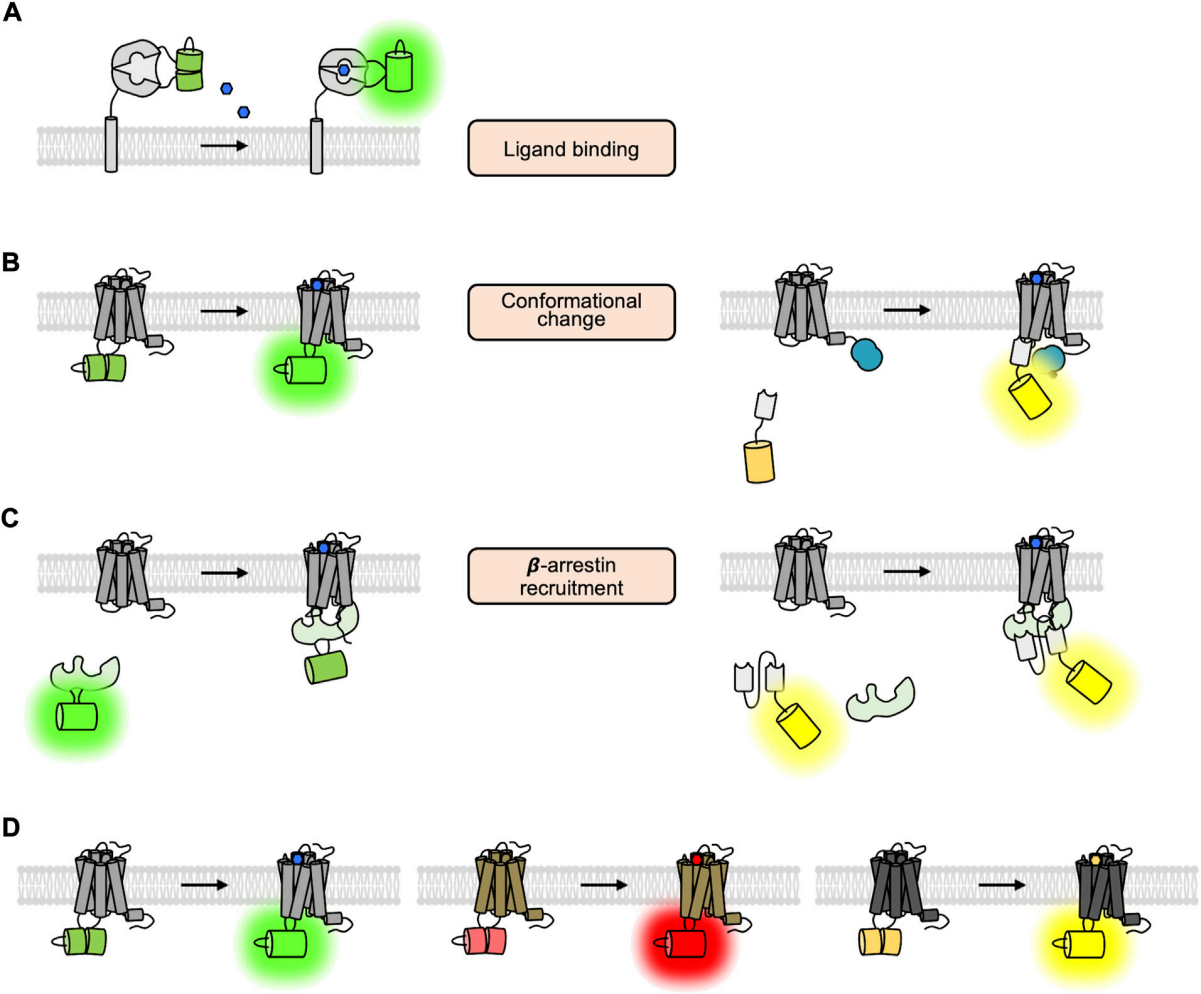
The GPCR biosensors utilizing cpFP and nanobody. **(A)** Schematic design of the ligand-sensing biosensors based on cpFP (Marvin et al., 2013). The fluorescent signal of cpFP inserted in the ligand-sensing domain is increased upon ligand binding. **(B)** Schematic design of fluorescent biosensors detecting the conformational change of GPCRs (Patriarchi et al., 2018; Sun et al., 2018). In the left panel, the fluorescent signal of the cpFP inserted in the ICL3 region of the GPCR is increased upon the conformational change of the GPCR. In the right panel, the YFP-tagged nanobody can specifically bind to the GPCR of active conformation. As a luciferase is fused to the GPCR, thus the BRET signal between the YFP and the luciferase is increased. **(C)** Schematic design of the cpFP- and nanobody-based biosensors detecting the recruitment of G proteins (Hoare et al., 2020). **(D)** Different color variants of cpFP-based GPCR biosensors (Patriarchi et al., 2020; Sun et al., 2020; Labouesse and Patriarchi, 2021).

To elucidate the molecular mechanisms of GPCR activation and function with high spatiotemporal resolution, a variety of strategies of GPCR biosensors based on fluorescent proteins (FPs) are developed, which allow the real-time monitoring of the GPCR activation in live cells (Alvarez-Curto et al., 2010; Lohse et al., 2012; Clister et al., 2015; Haider et al., 2019). First, the GPCR biosensors based on fluorescent resonance energy transfer (FRET) are designed to detect extracellular GPCR ligands (Okumoto et al., 2005; Hires et al., 2008; Zhang et al., 2018) (Figure 2A) and to monitor the conformational change of GPCRs upon the binding of the ligands (Stumpf and Hoffmann, 2016; Kauk and Hoffmann, 2018) (Figure 2B, left). The FRET technology is also utilized to detect the interaction between GPCRs and their downstream G proteins (Hein et al., 2005) (Figure 2C, left) or β-arrestin (Krasel et al., 2005) (Figure 2D, left), as well as GPCR oligomerization (Pfleger and Eidne, 2005) (Figure 2E). Similarly, bioluminescence resonance energy transfer (BRET) is also applied to monitor the recruitment of G proteins (Salahpour et al., 2012) (Figure 2C, middle and right) or β-arrestin to the activated GPCRs (Salahpour et al., 2012; Zhou et al., 2021) (Figure 2D, right, Table 1). More recently, a novel strategy of biosensors utilizing nanobodies has been developed to detect the active GPCR conformation or the recruitment of β-arrestin (Shukla et al., 2013; De Groof et al., 2019) (Figure 3B,C, right). In addition, the circular permuted FP-based GPCR biosensors are developed to detect the extracellular GPCR ligands (Marvin et al., 2013; Marvin et al., 2018; Dana et al., 2019; Marvin et al., 2019; Borden et al., 2020; Aggarwal et al., 2022), the conformational change of GPCR upon ligand binding (Labouesse and Patriarchi, 2021), or the recruitment of β-arrestin (Hoare et al., 2020) (Figure 3A–C, left).

**TABLE 1:** Examples of currently available FRET and BRET biosensors for GPCR studies.

Detection step	Target GPCR	Ligand used	Detection method	FRET or BRET pair	Cell lines tested	Notes	Reference
Ligand binding	GltI (mGluR)	Glutamate	FRET	ECFP-Venus	HEK293, HeLa		Hires et al., 2008 Tsien, 2006
Conformational change of GPCR	α2AAR	Noradrenaline, Norepinephrine	FRET	CFP-YFP, CFP-FlAsH	HEK293		Vilardaga et al., 2005 Nikolaev et al., 2006
	β1AR	Norepinephrine, Isoproterenol	FRET	Cerulean-YFP, Cerulean-FlAsH	HEK293		Rochais et al., 2007
	β2AR	Isoproterenol	FRET	CFP-YFP, CFP-FlAsH	HEK293		Reiner et al., 2010 Nakanishi et al., 2006
	A2AR	Adenosine	FRET	CFP-YFP, CFP-FlAsH	HEK293, COS-1		Hoffmann et al., 2005
	B1R	Carboxypeptidase M	FRET	CFP-FlAsH	HEK293		Zhang et al., 2013
	B2R	Bradykinin, Mechanical force	FRET	CFP-YFP	HEK293, BAEC		Chachisvilis et al., 2006
	H1R	Histamine, Mechanical force	FRET	Cerulean-FlAsH	HEK293		Erdogmus et al., 2019
	mAChR	Carbachol	FRET	CFP-FlAsH	HEK-TsA201		Maier-Peuschel et al., 2010
	PTH1R	PTH, Mechanical force	FRET	CFP-YFP, Cerulean-Citrine	HEK293, NC3T3-E1		Vilardaga et al., 2003, Zhang et al., 2009
	AT1R	Angiotensin II	BRET	RLuc-YFP	CHO		Szalai et al., 2012
	B1R	Carboxypeptidase M	BRET	RLuc-FlAsH	HEK293		Zhang et al., 2013
G protein recruitment	β2AR	Isoproterenol	FRET	mCerulean-mCitrine	HEK293		Malik et al., 2013
	V1AR	AVP	FRET	mCerulean-mCitrine	HEK293		Gupte et al., 2017
	A1AR	Adenosine	FRET	Cerulean-Venus	HEK293	Mini-G	Wan et al., 2018
	α2AAR	Brimonidine	BRET	NLuc-YFP	HEK293T	BERKY	Maziarz et al., 2020
	β2AR	Isoproterenol	BRET	RLuc-GFP	HEK293T		Gales et al., 2005
	β2AR	Isoproterenol	BRET	RLuc8-Venus	HEK293	Mini-G	Wan et al., 2018
	GLP-1R	GLP-1	BRET	NLuc-Venus	HEK293	Mini-G	Manchanda et al., 2021
β-arrestin recruitment	β2AR	Isoproterenol	FRET	CFP-FlAsH	HEK293		Nuber et al., 2016
	β2AR, V2R	Isoproterenol, AVP	BRET	RLuc-YFP	HEK293, COS		Charest et al., 2005
	β2AR, AT1R	Isoproterenol, Angiotensin II	BRET	NLuc-CyOFP1	HEK293T		Oishi et al., 2020
	PTH1R	PTH	BRET	RLuc-FlAsH	HEK293		Lee et al., 2016
GPCR dimerization	5HT2A/DRD2	DOI, Quinpirole	FRET	ECFP-EYFP	HEK293		Lukasiewicz et al., 2010
	mGluR1	Quisqualate	FRET	CFP-YFP	HEK293, COS-7		Hlavackova et al., 2012
	5HT2A/DRD2	5-HT, Quinpirole	BRET	RLuc-GFP2	HEK293T		Borroto-Escuela et al., 2010

GltI, Glutamate/aspartate import solute-binding protein; α2AAR, α2A adrenergic receptor; β1AR, β1 adrenergic receptor; β2AR, β2 adrenergic receptor; A2AR, A2A adenosine receptors; B1R, B1-bradykinin receptor; B2R, B2-bradykinin receptor; H1R, Histamine H1 receptor; mAChR, Muscarinic acetylcholine receptor; PTH1R, Parathyroid hormone 1 receptor; AT1R, Angiotensin II type 1 receptor; V1AR, Vasopressin receptor 1A; A1AR, A1A adenosine receptors; GLP-1R, Glucagon like peptide-1 receptor; V2R, Vasopressin receptor 2; 5HT2A, Serotonin receptor 2A; DRD2, Dopamine receptor 2; mGluR1, Metabotropic glutamate receptor 1; PTH, Parathyroid hormone; AVP, Arginine Vasopressin; GLP-1, Glucagon like peptide-1; DOI, 2,5-Dimethoxy-4-iodoamphetamine.

In this review, we present genetically encoded fluorescent biosensors that can reveal different stages of the GPCR activation with high spatiotemporal resolution. These stages include 1) binding extracellular ligand to GPCR, 2) conformational change of GPCRs, 3) recruitment with G proteins or β-arrestin, 4) GPCR internalization and trafficking, and 5) GPCR-related downstream signaling events. We will also cover the fluorescent biosensors to monitor the GPCR dimerization and oligomerization. The pros and cons of these GPCR biosensors will be discussed as well as our perspective on this research area.

## Binding extracellular ligand to GPCR

The first step of the GPCR activation is binding to its extracellular ligand (Figure 1A). The GPCR activation is initiated by the increased ligand concentrations in the extracellular space, thus the fluorescent biosensors detecting extracellular GPCR ligands have been designed whose response can indicate the initiation of the GPCR activation.

First, the FRET-based biosensors to detect extracellular GPCR ligands are composed of a ligand-binding protein interspersed with a FRET pair of FPs. They are presented outside of the plasma membrane, thus can change the FRET signals upon binding to the ligands (Figure 2A). We can monitor the increased ligand concentrations in live cells by detecting the FRET changes of the biosensors, which in turn will initiate the activation of GPCRs and intracellular signaling pathways. For example, the Tsien group developed the glutamate-sensing fluorescent reporter (GluSnFR) (Tsien, 2006) that is composed of a glutamate periplasmic binding protein, GltI, between cyan and yellow FPs in a pDisplay vector containing the TM domain of platelet-derived growth factor receptor (PDGFR). It has been shown that the improved version of GluSnFR, SuperGluSnFR, can report the level of glutamate during synaptic release, spillover, and reuptake in cultured hippocampal neurons (Hires et al., 2008). Similar design strategy can be applied to develop the fluorescent sensors for other ligands (Zhang et al., 2018).

The second design of the ligand-sensing fluorescent biosensors is composed of a circular permutated FP (cpFP) (Baird et al., 1999) inserted in a specific ligand-binding domain and located at the outside plasma membrane (Figure 3A). Upon binding of the ligand, the conformation of the ligand-binding domain is changed, which then stabilizes the chromophore of the cpFP, increasing its fluorescence intensity (Kostyuk et al., 2019). Thus, we can detect the increased concentration of the GPCR ligand by measuring the intensity of fluorescence of the biosensor and expect the subsequent activation of the GPCRs. Compared to the FRET-based biosensors utilizing two FPs (donor and acceptor), the cpFP-based biosensors based on a single FP are smaller in size, and the fold-change in fluorescence signals (∆F/F) is generally larger (∼250%) than the one of FRET-based biosensors (∼44%) (Kostyuk et al., 2019).

The representative example of the cp-based ligand-sensing fluorescent biosensor is iGluSnFR, which is composed of the glutamate binding domain GltI, an inserted cpEGFP, and the TM domain from PDGFR (Marvin et al., 2013). In addition to the cpEGFP-based green iGluSnFR, a red-colored R-iGluSnFR1 was developed, expanding the color palette of the glutamate biosensor (Wu et al., 2018). The second generation of iGluSnFR was generated by replacing EGFP with superfolder GFP (sfGFP), which shows the improved fluorescent signal change and a higher expression level (Marvin et al., 2018). Furthermore, different colors of SF-iGluSnFR are developed: SF-Azurite-iGluSnFR (blue), SF-iGluSnFR (green), and SF-Venus-iGluSnFR (yellow) (Marvin et al., 2018).

The third generation iGluSnFR3 was recently developed with further improved signal-to-noise ratios (∆F/F = ∼5,400 ± 260%) (Aggarwal et al., 2022). Different membrane-targeting strategies, in addition to the PDGFR TM domain, were applied, such as glycosylphosphatidylinositol (GPI) anchor and the cytosolic C-terminal domain of Stargazin thus iGluSnFR3 shows the better membrane trafficking and localization (Aggarwal et al., 2022). The ligand-sensing biosensors for other GPCR ligands have also been developed, for example, γ-aminobutyric acid (GABA), nicotine, and acetylcholine biosensors (Marvin et al., 2013; McManus et al., 2019; Borden et al., 2020). These GPCR biosensors enabled the real-time monitoring of the ligand release in cultured cells as well as *in vivo* (Sabatini and Tian, 2020).

## Conformational change of GPCR

### Ligand-induced GPCR activation

Upon binding to the ligands, GPCRs undergo conformational changes (Figure 1B), which can stabilize the interaction with heterotrimeric G proteins (Kobilka, 2007; Manglik and Kruse, 2017; Weis and Kobilka, 2018). The biosensors detecting GPCR ligands, explained above, inform the changes in the levels of extracellular GPCR ligands but cannot fully confirm the activation of GPCRs. Furthermore, the same ligand can activate different GPCR subtypes, which sometimes mediate opposite cellular outcomes (Wheatley et al., 2012; Wacker et al., 2017). For example, dopamine can bind to all five subtypes of dopamine receptors (DRD1 to DRD5), but the activated DRD subtypes induce different signaling pathways depending on the recruited G proteins. For example, the activated DRD1 activates adenylyl cyclase (AC) increasing cAMP in contrast the DRD2 activation inhibits AC decreasing cAMP (Neve et al., 2004; Undieh, 2010). Therefore, the fluorescent biosensors that detect the conformational changes of individual GPCRs, such as DRD1 and DRD2 biosensors, would be useful in understanding the dopamine-related signaling pathways through the different subtypes of DRD.

Crystal structures of GPCRs have revealed that the largest conformational change occurs between TM5 and TM6 during GPCR activation (Kobilka, 2007; Manglik and Kruse, 2017; Weis and Kobilka, 2018). These structural features are applied to design the FRET-based sensors that are fused to a donor FP in the C-terminus and an acceptor FP in the intracellular loop 3 (ICL3) between TM5 and TM6 of GPCRs (Figure 2B, left), for example, α2A adrenergic receptor (α2AAR) (Vilardaga et al., 2005), β1 adrenergic receptor (β1AR) (Rochais et al., 2007), β2 adrenergic receptor (β2AR) (Reiner et al., 2010), parathyroid hormone 1 receptor (PTH1R) (Vilardaga et al., 2003), and B2-bradykinin receptor (B2R) (Chachisvilis et al., 2006). Upon the binding to the ligands, the structural change between TM5 and TM6 of the biosensor induces the rearrangement of the acceptor FP inserted in the ICL3, causing the FRET changes. Thus, these FRET biosensors can report the conformational change of GPCRs upon activation in live cells.

Similarly, the FRET biosensors detecting the GPCR conformational change were developed based on donor FP and fluorescein arsenical hairpin binder (FlAsH) (Griffin et al., 1998). In this design, the sequence for the attachment of the FlAsH (i.e., CCPGCC) is encoded in the ICL3, and the donor FP (e.g., CFP) is fused to the C-terminus of GPCRs, for example, muscarinic acetylcholine receptor (mAChR) (Maier-Peuschel et al., 2010), α2AAR (Nikolaev et al., 2006), β2AR (Nakanishi et al., 2006), and A_2A_ adenosine receptors (A2AR) (Hoffmann et al., 2005). Thus, the FRET changes between CFP and FlAsH can report the ligand-induced conformational changes of these GPCRs in live cells. Because the chemical coupling reaction between FlAsH and the encoded motif sequence is required, this design is not completely genetically encodable (Hoffmann et al., 2010). On the other hand, the size of the fluorescent dye FlAsH is smaller than FPs (Griffin et al., 1998), thus this design of biosensor may mimic the natural structural states of the GPCR activation with less steric hindrance (Hoffmann et al., 2005). The donor FP in the C-terminus of the biosensor can be replaced with Renilla luciferase (RLuc) (Hastings, 1996) to generate the BRET-based biosensor, and the BRET changes between RLuc and the FlAsH can report the conformational change of GPCRs upon activation (Szalai et al., 2012; Zhang et al., 2013) (Figure 2B, right).

More recently, the cpFP-based biosensors detecting the conformational change between TM5 and TM6 of GPCRs have been developed (Figure 3B, left). The representative examples of this strategy are dLight and GRAB-DA, which are DRD biosensors developed by Tian and Li groups, respectively (Patriarchi et al., 2018; Sun et al., 2018). These DRD biosensors were constructed by inserting cpGFP into the ICL3 region of DRDs, and the conformational change of DRD can increase the fluorescent intensity of the inserted cpFP in the biosensor. From the optimization process in the linker sequences between GPCR and the inserted cpGFP, the dynamic range of these sensors was dramatically increased (∆F/F _dLight1.1_ = 230% and ∆F/F _GRAB-DA1m_ = 190%). As dLight and GRAB-DA can sensitively detect the dopamine-induced activation of DRDs, these DRD biosensors have been successfully applied to measure dopamine signals in various brain regions of mice (Kunishima et al., 2000; Patriarchi et al., 2018; Augustine et al., 2019; Lutas et al., 2019; Mohebi et al., 2019; Qian et al., 2019; Lefevre et al., 2020; Unger et al., 2020) as well as fly and zebrafish (Sun et al., 2018). We further developed a red-colored DRD1 sensor (R-DRD1) and a green-colored DRD2 sensor (G-DRD2), which can distinguish the activation of different DRD subtypes, i.e., DRD1 and DRD2, in the same cells (Kim et al., 2022) (Figure 3D). The cpFP-based biosensors that detect the conformational change of other GPCRs have also been developed (Feng et al., 2019; Jing et al., 2020; Patriarchi et al., 2020; Sun et al., 2020; Unger et al., 2020; Wan et al., 2021).

Another strategy to report the conformational change of GPCRs is based on nanobody (Nb) (Figure 3B, right). Nanobody is a recombinant single variable domain of a heavy chain fragment that was first isolated from the Camelidae family (Hamers-Casterman et al., 1993). While maintaining the ability for antigen binding, nanobody has useful biochemical properties such as tiny size, excellent solubility, and high stability (Muyldermans, 2013). The representative example of the Nb-based GPCR sensor is Nb80-GFP developed by the Zastrow group, which specifically binds to the active conformation of the β2AR (Rasmussen et al., 2011a). Nb80-GFP can visualize the β2AR activation by green fluorescent signals in live cells, which are displayed at the plasma membrane right after the treatment of an agonist for β2AR. Nb80-GFP further revealed the prolonged GFP signals at the internalized endosomes, providing direct evidence that β2ARs can keep their active conformation in endosomes after being internalized from the plasma membrane.

Another Nb-based GPCR sensor, Nb33-GFP, that specifically binds to the activated mu and delta opioid receptor (MOR and DOR), revealed a spatiotemporal landscape of OR activation in a ligand-dependent manner; peptide agonists activate ORs at the plasma membrane and internalized endosomes, in contrast, non-peptide drugs can activate a Golgi-localized internal OR pool (Stoeber et al., 2018). In addition to its specificity to GPCRs, these Nb-based biosensors can report the activation status of endogenous GPCRs without overexpression of exogenous GPCRs. On the other hand, the Nb-based biosensors may not be able to bind to the GPCRs surrounded by clathrin coats during the early stage of the internalization process (Irannejad et al., 2013), thus cannot report the status of GPCRs in this stage. Furthermore, Nb-based biosensors are designed to measure the activation state of specific GPCRs, thus these tools are not appropriate for research on multiple GPCRs.

### Mechanical stimulation-mediated GPCR activation

In addition to diverse GPCR ligands, some GPCRs respond to mechanical stimulation to mediate various physiological processes. For example, in the vascular endothelial cells of small-diameter arteries, fluid shear stress was identified as a mechanical stimulation through mechanosensitive G protein-coupled receptor 68 (GPR68), which is involved in the local regulation of vascular resistance (Xu et al., 2018). However, the precise mechanism by which mechanosensitive GPCRs transduce mechanical stimuli into intracellular responses remains unclear. Thus, the investigation of the conformational dynamics of mechanosensitive GPCRs upon mechanical stimulus is important to understand their roles in physiology and pathophysiology.

The FRET technique was applied to visualize the conformational changes of mechanosensitive GPCRs upon mechanical stimulations such as fluid shear stress and hypotonic membrane stretch. For example, B2R (Chachisvilis et al., 2006) and PTH1R (Zhang et al., 2009) were demonstrated to be mechanosensitive by intra-molecular FRET biosensors that are constructed by inserting YFP into the ICL3 and fusing CFP to the C terminus (Figure 2B, left). In addition, the series of FlAsH-based FRET sensors, which are encoded with the FlAsH motif in different regions of GPCRs, revealed the mechanical force-mediated elongation of the helix 8 (H8) domain of GPCRs might be essential for the mechanosensitive response (Erdogmus et al., 2019).

In addition, the cpFP-based biosensor iGlow was developed to detect the mechanical stimulation-induced conformational change of a GPR68 (Ozkan et al., 2021). (Figure 3B, left). It was generated by inserting cpGFP into the ICL3 of GPR68, thus increasing its fluorescent intensity in response to fluid shear stress. The dynamic range of iGlow is from 25% to a maximum of 75% under a single fluid shear stress pulse between 10.4 and 20.8 dyne/cm^2^. Further investigation using this strategy of biosensors will provide more information on the mechanosensitive GPCRs.

## G protein coupling to GPCR

The next step of the GPCR signaling pathway after the conformational change of the receptor is the coupling of heterotrimeric G proteins to the GPCR (Figure 1C). The recruited G proteins to the GPCRs then initiate the downstream signaling events, thus fluorescent biosensors for detecting the recruitment of G proteins are developed utilizing the following strategies.

The first strategy is to directly visualize the translocation of the FP-tagged G alpha protein to GPCR to the plasma membrane (Hynes et al., 2004). In addition, the inter-FRET signals between the G alpha protein tagged with a donor FP, and the GPCR fused to an acceptor FP can be measured to quantify the levels of G protein coupling to the GPCR (Hein et al., 2005). Similarly, the donor FP can be replaced with luciferase, and thus the inter-BRET signals can report the coupling between the G protein and activated GPCR (Gales et al., 2005). However, these approaches include the overexpression of G proteins (Azzi et al., 2001; Janssen et al., 2002), and require the expression of the heterotrimeric form of G proteins (G alpha, beta, and gamma) for the correct localization at the plasma membrane (Rasmussen et al., 2011b; Rosenbaum et al., 2011) may influence higher basal levels of the G protein-related downstream pathways.

Mini-G is the engineered G alpha protein that contains only essential sequences for the coupling to the GPCR (Oldham et al., 2006; Carpenter and Tate, 2016). To construct the mini-Gs, three regions of Gs, i.e., GαAH, switch III, and half of the N-terminal helix, were deleted and several mutations were applied to the residues in the nucleotide-binding pocket, switch II, and the α5 helix (Carpenter and Tate, 2016). Different versions of mini-G, i.e., mini-Gs, mini-Gi, mini-Gq, and mini-G12, have been further developed to detect the coupling of different G proteins (i.e., G alpha s, i, q, 12) to the relevant GPCRs (Nehme et al., 2017). Mini-G can be fused to FP for the direct monitoring of the translocation to the plasma membrane in response to ligands for the target GPCRs; in addition, mini-G and the target GPCR are fused to FPs or luciferases, and the interaction between specific G proteins and target GPCRs can be measured by FRET or BRET signals (Wan et al., 2018; Manchanda et al., 2021) (Figure 2C). The overexpression of these biosensors, however, may perturb endogenous G protein downstream signaling pathways, thus they can be optimally used to monitor and measure the step of the G protein recruitment.

The second sensing strategy to detect the G protein coupling to the receptor is the intra-molecular FRET-based GPCR sensor which is composed of GPCR, FRET pair FPs between ER/K linker (Sivaramakrishnan and Spudich, 2011), and short C-terminal sequences from the alpha helix 5 of G alpha protein (Figure 2C, left). During the coupling of the G alpha protein to the activated GPCR, the selection of G alpha protein, e.g., G alpha s, i/o, q, 12/13, is dependent on the direct interaction between the GPCR and the C-terminus sequences of the G alpha protein (Semack et al., 2016; Tsai et al., 2019). Thus, the alpha helix 5 sequences of each G protein are crucial to determine the binding specificity to relevant GPCRs (Flock et al., 2017; Inoue et al., 2019a).

For example, β2AR-Gs FRET biosensor was developed which contains β2AR, cyan and yellow FPs, and the C-terminal 27 residues from the alpha helix 5 of Gs (Malik et al., 2013). The ER/K linker, which is a subset of single alpha-helical domains that contains 73 amino acids (Sivaramakrishnan and Spudich, 2011), enables the efficient separation between the donor and acceptor FPs to keep the low FRET level in the default state. When the activated β2AR in the biosensor undergoes the conformational change, the C-terminal sequences derived from Gs can directly bind to the β2AR in the biosensor. This increases the proximity between the donor and acceptor FPs in the β2AR-Gs biosensor, thus the increased FRET level can report the G protein coupling to the activated GPCR in live cells.

Utilizing these FRET-based biosensors, the structural basis of selective interactions between GPCR and the C-terminus of G alpha protein was further investigated (Semack et al., 2016). In particular, the FRET-based β2AR-Gs biosensor and vasopressin receptor 1A (V1AR)-Gq biosensor were constructed. The FRET measurement discovered three hot spot residues in the C-terminus of G alpha protein that are critical for determining the coupling specificity of G protein. Additional mutation experiments in these residues further revealed that electrostatic interaction is important for the β2AR-Gs complex, whereas the V1AR-Gq interface is predominantly hydrophobic (Semack et al., 2016), suggesting different coupling mechanisms of Gs and Gq to relevant GPCRs. In addition, another study utilizing these biosensors revealed that non-cognate G alpha proteins could modulate the ligand-binding affinity of the GPCR through allosteric interaction or different binding modes to the receptor (Gupte et al., 2017).

Finally, the third strategy is the BRET-based detection of endogenous interaction between G proteins to GPCRs via the specific detector module that binds to the activated G proteins (Figure 2C, middle). This BRET-based biosensor with ER/K linker and YFP (BERKY biosensor) is composed of membrane anchoring sequence, NanoLuc, ER/K linker, YFP, and KB-1753 that is a synthetic peptide specifically binding to Gαi-GTP (Maziarz et al., 2020). In the default state, the ER/K linker in the Gαi-BERKY biosensor efficiently separates the donor and acceptor of BRET, minimizing the basal BRET level. When endogenous GPCRs are activated and coupled to Gαi-GTP, the KB-1753 in the Gαi-BERKY biosensor binds to the endogenous Gαi-GTP proteins coupled to GPCR. This allows the ER/K linker to bring the donor and acceptor proximal to each other, resulting in an increased BRET signal. In addition, the BERKY sensors for Gαq-GTP, Gα13-GTP, free Gβγ and Rho-GTP were further developed by replacing the G protein-specific module (Maziarz et al., 2020). Thus, the endogenous GPCR-G protein coupling event can be monitored by the BERKY biosensor in live cells.

## Recruitment of β-arrestin to GPCR

After the activation of the G protein-mediated signaling pathway, the C-terminus sequences of the GPCR can be phosphorylated by G protein-coupled receptor kinases (GRKs) which in turn can recruit β-arrestins (Figure 1D). This prevents the G protein-mediated signal transduction by sterically hindering G protein coupling (Lefkowitz and Shenoy, 2005). Although some GPCRs can be internalized through arrestin-independent mechanisms (Moo et al., 2021), it is known that the β-arrestin facilitates the internalization of GPCR via interaction with clathrin and adaptor protein 2 (AP2). (Goodman et al., 1996; Laporte et al., 1999). In addition to this classical role of β-arrestin for GPCR internalization, emerging evidence suggests that β-arrestin can mediate signaling cascades via G protein-independent pathways (Lefkowitz and Shenoy, 2005) (Figure 1D). For example, β-arrestin can serve as a scaffolding protein for mitogen-activated protein kinases (MAPKs) cascades, which regulate various cellular functions, including proliferation, transcriptional regulation, and apoptosis (DeWire et al., 2007).

For such a different function, β-arrestin may adopt multiple conformations and be modulated at various levels (Shukla et al., 2008). Different β-arrestin conformations include 1) “tail” conformation, with β-arrestin coupled to the phosphorylated C-terminal tail of GPCR, and 2) “core” conformation, where β-arrestin interacts with the receptor TM core region through its finger-loop region (Shukla et al., 2014). Because GPCRs may exist in multiple conformational states, the ligands which can selectively stabilize different conformational states of the GPCRs can recruit distinct effector proteins, initiating G protein- or β-arrestin-mediated signaling pathways (Kobilka and Deupi, 2007). This functional selectivity, also called biased agonism, allows the selective targeting of beneficial pathways while avoiding potentially detrimental ones (Wootten et al., 2018). Considering the critical roles of β-arrestin in various cellular events, it is necessary to develop fluorescent biosensors for monitoring the formation of the GPCR-β-arrestin complex or visualizing the active conformation of β-arrestin in live cells.

First, intra-molecular BRET-based biosensors were constructed to probe conformational change of β-arrestin by fusing RLuc and YFP to the N- and C-terminus of β-arrestin respectively (Charest et al., 2005) (Figure 2D, right). In addition, GFP was fused to the C-terminus of GPCR to assess whether RLuc-β-arrestin-YFP is recruited to the agonist-activated GPCR, and the intermolecular BRET signals between RLuc and GFP reflect the recruitment of RLuc-β-arrestin-YFP to the GPCR. In a similar way, another intra-molecular BRET biosensor was developed with NanoLuc and CyOFP1 as a donor and an acceptor, respectively. Nanoluc has a smaller size (19 kDa) compared to RLuc (36 kDa) with an improved brightness (∼150-fold) (Hall et al., 2012), and CyOFP1 is a cyan-excitable orange FP (Oishi et al., 2020). This β-arrestin biosensor allows the detection of different conformations of β-arrestin associated with GPCR, i.e., tail and core conformations.

In addition, the FlAsH-based intra-molecular FRET/BRET biosensors were developed to predict more precisely the conformational changes of β-arrestin during activation, e.g. a BRET-based biosensor based on FlAsH-RLuc pair (Lee et al., 2016) and a FRET-based biosensor with FlAsH-CFP pair (Nuber et al., 2016) (Figure 2D, left). These biosensors were generated as a series of different biosensors in which the FlAsH motif was encoded at different regions of β-arrestin. First, the BRET-based β-arrestin biosensors with six different FlAsH sites were examined with various GPCRs. The results revealed the conserved features of BRET signals between GPCRs for controlling the ERK1/2 phosphorylation, indicating a distinctive β-arrestin conformational signature for the β-arrestin-dependent downstream signaling events. In addition, the FRET-based β-arrestin biosensors with eight different FlAsH sites were used to investigate the kinetics in the conformational changes of β-arrestin during the coupling and uncoupling between β-arrestin and GPCRs.

Finally, the single FP-based β-arrestin biosensor was engineered by inserting the entire β-arrestin into the critical seventh stave of the β-sheet directly adjacent to the chromophore of mNeonGreen (Hoare et al., 2020) (Figure 3C, left). Interestingly, mNeonGreen in this biosensor is not circularly permuted, thus it has a high quantum yield allowing the biosensor to read sufficient signals with a brief excitation time and thereby minimizing photobleaching (Shaner et al., 2013). When β-arrestin binds to the phosphorylated GPCR, the conformational rearrangement in the biosensor changes the chromophore environment, resulting in a decrease in fluorescence intensity. Due to its high temporal resolution and minimal photobleaching, the biosensor can be used to measure the dynamic kinetics of the arrestin-dependent signaling pathway.

## GPCR signaling at internalized endosomes and other organelles

Previous studies focus on GPCR activity at or near the plasma membrane, where the GPCRs are known to mainly function in response to extracellular stimuli. Likewise, it has been believed that GPCRs transmit these signals only at the plasma membrane and are desensitized after internalization (Ferguson, 2001). However, emerging evidence suggests that some GPCRs can maintain their activity at endosomes or subcellular organelles even after internalization (Eichel and von Zastrow, 2018) (Figure 1E). For example, the internalized β2AR can continue its function at endosomes, which is crucial for altering the expression patterns of cAMP-dependent transcriptional factors (Tsvetanova and von Zastrow, 2014). Therefore, it is important to develop fluorescent biosensors which can monitor the real-time activity of GPCRs at subcellular organelles in live cells.

The sustained GPCR activity at the internalized endosomes was visualized by Nb-based fluorescent biosensors. As mentioned in the previous section, the Zastrow group showed that β2ARs could keep their active conformation at the internalized endosomes utilizing Nb80-GFP (Irannejad et al., 2013). It was also revealed, using a nanobody-based biosensor Nb80-FP, that the β1AR-induced G protein signaling occurs in the Golgi, suggesting novel pharmacological approaches for the treatment of heart failure (Nash et al., 2019). In addition, Nb37-FP showed that the internalized Thyroid stimulating hormone (TSH) receptor maintains its function in the Golgi and Trans-Golgi network (TGN) (Godbole et al., 2017). This sustained activity of TSH produces cAMP near the nucleus, which can efficiently induce the phosphorylation of cAMP response element-binding protein (CREB) (Godbole et al., 2017). The Nb-based OR sensor Nb33-GFP also discovered the Golgi-localized internal OR pool which can be activated by non-peptide drugs, while peptide agonists activate ORs conventionally at the plasma membrane and internalized endosomes (Stoeber et al., 2018). These studies using the nanobody-based fluorescent biosensors provide strong evidence that GPCRs maintain their functions at internalized endosomes and other organelles.

**FIGURE 4:**
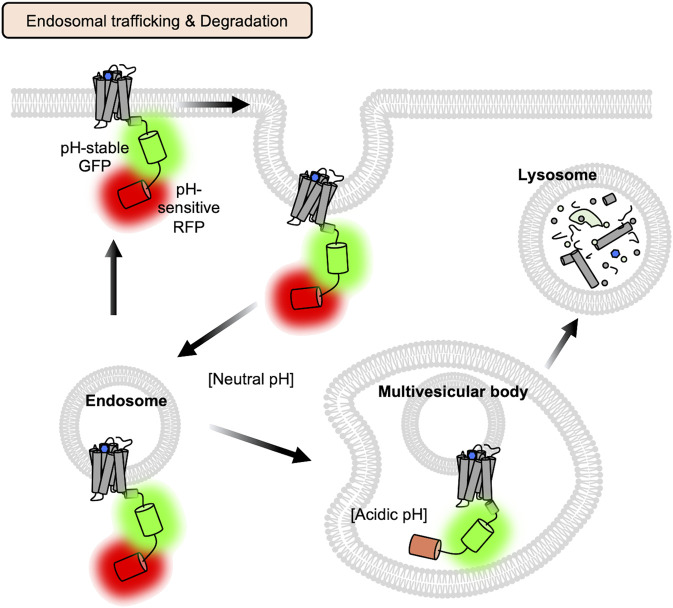
Monitoring endosomal trafficking and degradation process of GPCRs with the GPCR-pH sensor. To confirm that GPCRs encounter the more acidic environment during the endosomal trafficking and degradation process (Marchese et al., 2008; Kim et al., 2021a), a GPCR-pH sensor is designed, which is composed of β2AR, a pH-sensitive FP (Bizzarri et al., 2009; Shen et al., 2014) and a pH-stable reference FP, which can confirm that GPCRs encounter acidic environment during endosomal trafficking pathways. Acidification in the endosomes and lysosomes decreases the fluorescent intensity of the pH-sensitive RFP, but not the reference FP, thus the acidic state of GPCRs can be predicted by the intensity ratios of pH sensitive-FP (red) and reference FP (green).

For the real-time monitoring of GPCR activation kinetics during endosomal trafficking and degradation pathways (Figure 1F), we conducted long-term live-cell imaging of the FRET-based β2AR biosensor, which contains β2AR, a FRET pair FPs, and the C-terminal sequences from Gs (Kim et al., 2021a) (Figure 2C, left). As sensitive and reversible FRET signals can report the real-time status of β2AR activity during the entire process of the endosomal trafficking pathway, we can confirm that the internalized β2AR is active at the endosomes for several hours before it is degraded (Kim et al., 2021a). We further calculated the kinetic factors of β2AR activation during endosomal trafficking and degradation pathways, which would be useful for assessing the functional kinetics of particular drugs on target GPCRs. We also designed a GPCR-pH sensor which is composed of β2AR, a pH-sensitive FP (Bizzarri et al., 2009; Shen et al., 2014) and a pH-stable reference FP, which can confirm that GPCRs encounter an acidic environment during endosomal trafficking pathways (Marchese et al., 2008; Kim et al., 2021a) (Figure 4).

## Dimerization or oligomerization of GPCR

Emerging evidence suggests that some GPCRs can be dimerized or oligomerized (Hebert and Bouvier, 1998; Fuxe et al., 2010; Ferre et al., 2014) (Figure 1G). Functional crosstalk between these GPCRs may influence the ligand binding affinity, downstream signaling pathways, and trafficking patterns (Jordan and Devi, 1999; Casado et al., 2007; Fiorentini et al., 2008; Milligan, 2010; Gonzalez-Maeso, 2011; Gomes et al., 2016), and some GPCR oligomerization populations may be related to disease states (Ferre et al., 1991; Jordan et al., 2003; Gonzalez-Maeso et al., 2008). However, previous GPCR studies assumed that each GPCR generally exists as a monomer. Therefore, it is important to monitor the status of dimerization or oligomerization of GPCRs and to detect functional crosstalk between the GPCR dimers or oligomers.

First, FRET or BRET techniques can be applied to identify the GPCR dimers or oligomers. For example, the physical interaction between DRD2 and serotonin receptor 2A (5HT2A) was demonstrated by analyzing inter-FRET signals between 5HT2A-CFP and DRD2-YFP (Lukasiewicz et al., 2010) (Figure 2E). In addition, the functional crosstalk between 5HT2A and DRD2 was first revealed through analysis of the inter-BRET signal between DRD2-RLuc and 5HT2A-GFP upon stimulation of selective agonist or antagonist of each receptor (Borroto-Escuela et al., 2010). As FRET and BRET can occur when the donor and the acceptor are proximal within 10 nm, it is generally believed that the attached molecules may interact closely each other (Padilla-Parra and Tramier, 2012). Nevertheless, other biochemical methods such as co-immunoprecipitation and PLA assay would be helpful to confirm the formation of the GPCR complexes (Faron-Gorecka et al., 2019).

Studies on homodimers were also conducted using FRET technology. For example, metabotropic glutamate receptor 1 (mGluR1) is known to form a homodimer when activated (Kunishima et al., 2000). However, the molecular sequence of homodimerization and the conformational change was not clear, thus inter-FRET and intra-FRET mGluR1 biosensors were constructed (Hlavackova et al., 2012). For the monitoring of the homodimerization process of mGluR1, subunits of inter-molecular FRET biosensors were constructed by fusing CFP or YFP to ICL2 of mGluR1. On the other hand, intra-molecular FRET sensor was developed by fusing YFP to ICL2 and CFP to the C-terminus for measuring conformational changes of mGluR1. The results utilizing these FRET sensors revealed that, upon ligand binding, the homodimerization of mGluR1 through the Venus flytrap (VFT) domain occurs first, and then the conformational changes in the ICL of mGluR1 follow next to induce the G protein-mediated signaling pathways. Thus, FRET-based biosensors are useful to elucidate the molecular mechanism of the GPCR activation process.

Functional crosstalk between GPCR heterodimers can also be investigated by cpFP-based biosensors. For example, we developed the cpFP-based red DRD1 biosensor and green DRD2 biosensor, R-DRD1 and G-DRD2, which selectively increase red or green fluorescence upon the activation of DRD1 or DRD2, respectively (Kim et al., 2022). The R-DRD1 biosensor was co-expressed with DRD2 to form the DRD1-DRD2 heterodimer (Hasbi et al., 2009; Urizar et al., 2011; Chun et al., 2013), then the activation status of DRD1 in the heterodimer was closely monitored in live cells. Also, G-DRD2 co-expressed with DRD1 can report the activation status of DRD2 in the heterodimer. The results of this study demonstrated that DRD1 in the DRD1-DRD2 heterodimer is selectively inhibited in response to high levels of dopamine, whereas DRD2 is inhibited only upon low levels of dopamine, suggesting differential functional crosstalk in the DRD1-DRD2 heterodimer under different physiological conditions (Kim et al., 2022). These methods utilizing fluorescent GPCR biosensors can be further applied to investigate functional crosstalk in other GPCR dimers and oligomers, promising a deeper understanding of the molecular mechanism of GPCR activation and function.

## GPCR downstream signaling

We have discussed a variety of fluorescent sensors to monitor the different status of the GPCR activation process, for example, the binding of an extracellular ligand to GPCR, conformational change of GPCRs, recruitment with G proteins or β-arrestin, GPCR internalization and trafficking, and GPCR dimerization and oligomerization (Figure 1). The final step of GPCR activation initiates the intracellular signaling events specific to the recruited G proteins.

First, the activation of GPCR coupled to Gs protein induces the production of cAMP through the activation of adenylyl cyclase (AC), while the activated GPCR coupled to Gi protein inhibits AC, thus reducing the level of cAMP (Sunahara and Taussig, 2002; Moreira, 2014). The cAMP level plays an important role in controlling protein kinase A (PKA) activity, which is crucial for CREB-mediated transcription (Delghandi et al., 2005). Therefore, it is useful to apply fluorescent sensors detecting cAMP level or PKA activation for monitoring the downstream functions of GPCRs coupled to Gs and Gi protein. An excellent review of these biosensors is available for further reading (Massengill et al., 2021).

Second, the activation of GPCRs coupled to Gq protein increases intracellular Ca^2+^ levels through the phospholipase C (PLC) signaling pathway (Smrcka et al., 1991; Taylor et al., 1991; Moreira, 2014). Because Ca^2+^ is an important second messenger for a wide variety of cellular processes, there are continuous efforts to develop efficient and sensitive Ca^2+^ sensors. The first FRET-based Ca^2+^ sensors were developed by the Tsien group, which can measure the FRET changes between a donor and an acceptor FP induced by the interaction of Ca^2+^-bound M13 and calmodulin (Miyawaki et al., 1997). This interaction was also applied to design the first cpFP-based Ca^2+^ sensors GCaMP (Nakai et al., 2001), which has been continuously improved for better sensitivity, brightness, stability, signal-to-noise ratio (Chen et al., 2013; Dana et al., 2019), and different colors (Zhao et al., 2011; Inoue et al., 2019b; Qian et al., 2019). Excellent reviews for the Ca^2+^ sensors are available (Kerruth et al., 2019; Li and Saha, 2021; Lohr et al., 2021).

Finally, the activation of G_12/13_-coupled GPCRs can induce the activation of Rho GTPases (Dhanasekaran and Dermott, 1996; Kozasa et al., 1998). Genetically encoded fluorescent sensors have been developed to monitor the activation of Rho GTPases in live cells based on FRET and dimerization-dependent FP (ddFP) (Kim et al., 2019). More information on the Rho biosensors is available in the previous review (Kim et al., 2021b). These biosensors detecting the GPCR downstream signaling can report the real-time functional outputs of GPCR activation, thus can be applied sequentially or together with other GPCR biosensors discussed in the previous sections.

## Conclusion

Upon the binding of extracellular ligands, GPCRs change their conformations to recruit G proteins and/or β-arrestin and mediate downstream signaling events. Then the GPCRs can be internalized and follow endosomal trafficking pathways for recycling or degradation (Figure 1). In this review, we discussed various strategies of genetically encoded fluorescent biosensors that can monitor these stages of GPCR activation with high spatiotemporal resolution. For example, the FRET/BRET-based GPCR biosensors have been designed to monitor different steps of GPCR activation (Figure 2). We discussed the circular permuted FP-based GPCR biosensors, which generally show higher dynamic ranges compared to the FRET/BRET-based biosensors (Figure 3). We also introduced nanobody-based GPCR biosensors (Figure 3) and the GPCR-pH sensor utilizing pH-sensitive FPs (Figure 4). These genetically encoded fluorescent biosensors can be applied to a wide range of GPCR-related research, therefore researchers need to choose the most appropriate GPCR biosensors for their research.

First, the GPCR biosensors are used to elucidate precise molecular mechanisms of GPCR activation and function. For example, the FRET-based biosensors that detect the conformational change of GPCRs provided important information regarding the structural rearrangement of GPCRs during activation (Erdogmus et al., 2019). The sustained GPCR activity at the internalized endosomes (Rasmussen et al., 2011a) as well as other organelles (Godbole et al., 2017; Stoeber et al., 2018) were revealed by Nb-based GPCR biosensors. The fluorescent biosensors that detect the coupling of G protein to GPCRs discovered the structural basis of selective interactions between GPCR and different G proteins (Semack et al., 2016). The dimerization of GPCRs can be identified by FRET-based biosensors (Hlavackova et al., 2012), and functional crosstalk between GPCR dimers can be assessed by cpFP-based GPCR biosensors (Kim et al., 2022). We expect that the development of novel GPCR biosensors and their applications with advanced imaging techniques will promise further exciting discoveries on the molecular mechanisms of GPCR activation and function.

Second, the GPCR biosensors can be used to sensitively monitor the activation of GPCRs in live cells and *in vivo*. Genetically encoded fluorescent biosensors are particularly useful in neuroscience due to their ability to visualize the spatiotemporal activity of neurotransmitter receptors, which are also GPCRs, in complex brain networks. For example, the cpFP-based biosensors were successfully used to detect the release of neurotransmitters such as glutamate (Hires et al., 2008) and GABA (Marvin et al., 2019). Moreover, the real-time activity of neurotransmitter receptors, for example, dopamine receptors (Patriarchi et al., 2018; Sun et al., 2018), was monitored in the mouse brain with the cpFP-based GPCR biosensors. For the understanding of complex brain functions, further efforts will be required to develop improved fluorescent biosensors with enhanced fluorescent response and higher expression levels. In addition to genetically encoded GPCR biosensors, the optogenetic technology which can spatiotemporally control the activity of GPCR, such as OptoXRs (Airan et al., 2009; Tichy et al., 2019), will be further useful to investigate the function of neurotransmission in the brain.

Finally, the GPCR biosensors can be applied for the screening of the drug candidates targeting GPCRs. As GPCRs are major therapeutic targets of more than 30% of FDA-approved drugs (Insel et al., 2019), the genetically encoded GPCR biosensors can be a useful platform for drug screening (Unger et al., 2020). The efficient agonists can be screened and selected by quantifying the fluorescent signals of GPCR biosensors in live cells. For the selected candidates, the functional kinetics of the agonist-induced GPCR activation can be accessed by the GPCR biosensors (Kim et al., 2021a). Furthermore, the biased agonism can be tested and characterized by the β-arrestin biosensors (Shukla et al., 2008; Hoare et al., 2020). Therefore, the genetically encoded GPCR biosensors are powerful tools to screen and evaluate the drug candidates targeting GPCRs.
